# Isolation and characterization of a novel lytic phage K14-2 infecting diverse species of the genus *Klebsiella* and *Raoultella*

**DOI:** 10.3389/fmicb.2024.1491516

**Published:** 2025-01-17

**Authors:** Seomin Kang, Jeong-Eun Han, Young-Sik Choi, In-Chul Jeong, Jin-Woo Bae

**Affiliations:** ^1^Department of Biology, College of Science, Kyung Hee University, Seoul, Republic of Korea; ^2^Department of Biomedical and Pharmaceutical Sciences, Kyung Hee University, Seoul, Republic of Korea; ^3^Department of Life and Nanopharmaceutical Sciences, Kyung Hee University, Seoul, Republic of Korea

**Keywords:** bacteriophage, phage, *Klebsiella pneumoniae*, *Klebsiella*, *Raoultella*, *Slopekvirus*

## Abstract

Multi-drug-resistant bacteria pose a significant global threat to public health, particularly among patients with critical nosocomial infection. Notably, the genera *Klebsiella* and *Raoultella* are of concern due to their association with human infections and the transfer of antibiotic-resistance genes. Phage therapy has recently garnered attention as a novel approach to treating these infections. However, the efficacy of this method relies on phages with a broad host range. In this study, the phage K14-2 with a wide host range was isolated from a river water sample using *Klebsiella pneumoniae* as the host. The biological properties of the phage were characterized by assessing its multiplicity of infection, killing curve, one-step growth curve, and stability across different pH levels and temperatures. The morphological analysis revealed that the phage closely resembled myovirus. The host range included 15 strains across 6 species from *Klebsiella*, *Raoultella*, and *Escherichia* genera. The genome of K14-2 was found to be double-stranded DNA, comprising 175,759 base pairs with a GC content of 41.8%. Genome annotation revealed 280 protein-coding genes, of which 96 had assigned functions. The phage with the highest genomic similarity to K14-2 was vB_KpM-Milk. Phylogenetic trees constructed based on the major capsid protein revealed that the phage belonged to the genus *Slopekvirus* of the Straboviridae family. Given these characteristics, the discovery of the novel phage K14-2, with its broad host range, holds potential for enhancing the effectiveness of phage therapy in future studies.

## 1 Introduction

In recent years, antimicrobial-resistant (AMR) bacteria have emerged as a significant threat to global public health (Antimicrobial Resistance Collaborators, 2022; Furuya and Lowy, 2006). The development of modern medicine has enabled the treatment of most infectious diseases with antibiotics. However, the widespread use of antibiotics has led to the overuse and misuse of antibiotics, resulting in the emergence of AMR bacteria and multidrug-resistant strains, commonly referred to as “superbugs” (Reygaert, 2018; Tenover, 2006). These superbugs are particularly dangerous, causing severe nosocomial infections in immunocompromised patients and posing a serious threat to their lives.

The genus *Klebsiella* is well-known for its association with human infections and diseases (Dong et al., 2022; Podschun and Ullmann, 1998). Similarly, *Raoultella*, a genus closely related to *Klebsiella*, also infects humans, albeit with relatively lower virulence (Appel et al., 2021; Sekowska, 2017). Both genera are opportunistic pathogens commonly found in the human mucosa and the environment. Particularly, *Klebsiella pneumoniae*, a member of the ESKAPE group, which includes six pathogenic AMR bacteria, is well-known for causing a wide range of human diseases, including pneumonia, urinary tract infections, wound infections, bacteremia, and sepsis (Paczosa and Mecsas, 2016; Pitout et al., 2015). Beyond their disease-causing potential, these genera present an additional challenge owing to their ability to readily acquire drug resistance through the transfer of AMR genes via plasmids (Navon-Venezia et al., 2017; Ramirez et al., 2019). The acquisition of these genes complicates treatment, underscoring the urgent need for new and effective treatment strategies.

Currently, various approaches, including bacteriophage (phage) therapy, antimicrobial peptides, antibodies, and microbiota-based therapeutics, are under development to eradicate AMR bacteria (Brooks and Brooks, 2014). Among these, recent studies on phage therapy have shown promising results (Chan et al., 2013; Gordillo Altamirano and Barr, 2019). To advance phage therapy research, a diverse array of lytic phages targeting different receptors used by various phages for infection is essential (Hyman, 2019; Ly-Chatain, 2014). However, the exploration of phage taxonomic diversity and characteristics remains in its early stages.

To address this gap in the literature, this study aimed to characterize a lytic phage, K14-2, from Jungnangcheon River water sample using *K. pneumoniae* as the host. Biological characteristics of the phage, including its multiplicity of infection (MOI), killing curve, one-step growth curve, physical stability, host range, and virion morphology were examined. Additionally, the phage genome was assembled and annotated to identify phage genes. Our findings indicate that phage K14-2 is a novel member of the genus *Slopekvirus*, capable of infecting a wide range of species within the *Klebsiella* and *Raoultella* genera.

## 2 Materials and methods

### 2.1 Bacterial strains and culture conditions

The type strain of *K. pneumoniae* subsp. *pneumoniae* KCTC 12385*^T^* was used as the host bacterium for bacteriophage isolation. For the host range assay, 21 bacterial strains were tested. These included *K. pneumoniae* subspecies, *Klebsiella alba*, *Klebsiella michiganensis*, *Raoultella ornithinolytica*, *Raoultella planticola*, *Acinetobacter baumannii*, *Escherichia coli*, *Pseudomonas aeruginosa*, and *Staphylococcus aureus*. Some of the biological resources used in this research were obtained from the Korean Collection for Type Cultures (KCTC) or the Korean Agricultural Culture Collection (KACC), and the remaining strains were isolated from various samples (Table 1). Bacterial species were identified through 16S rRNA gene sequencing, with sequences compared against the EzBioCloud database (Chalita et al., 2024). The genes were sequenced using the bacterial-specific universal primers 27F, 337F, 785F, 800R, and 1492R. The sequenced fragments were assembled to full-length 16 rRNA gene sequences with SeqMan 5.0 program (DNASTAR) (Swindell and Plasterer, 1997). To identify the relationship between the used bacterial species, a phylogenetic tree was constructed using the sequences. The sequences were aligned by CLUSTRAL W software in BioEdit v5.0.9 (Thompson et al., 1994). Neighbor-joining (NJ), maximum-likelihood (ML), and maximum-parsimony (MP) algorithms were used with 1,000 bootstrap replications for tree construction using the MEGA v7.0.26 program (Kumar et al., 2016). Also, for better understanding of the taxonomical positioning of the genus *Raoultella*, a core bacterial gene set was extracted from the reference genomes of the species from the genera *Klebsiella* and *Raoultella*. The genomes were acquired from NCBI and reconstructed to a phylogenetic tree by UBCG (Up-to-date Bacterial Core Genes) v3.0 software (Na et al., 2018). Bacterial cultures were incubated in liquid Luria-Bertani (LB, BD Difco) medium or on solid LB medium containing 1.5% (w/v) agar at 37°C in an incubator.

**TABLE 1:** Bacterial strains used for host range test.

Bacterial species	Strains	Lysis	Source
*Klebsiella pneumoniae* subsp. *pneumoniae*	KCTC 12385^T^	4	KCTC
	KCTC 2619	2	KCTC
	KCTC 2690	2	KCTC
*Klebsiella pneumoniae* subsp. *ozaenae*	KACC 22683	4	KACC
	KCTC 22058	0	KCTC
	KpM1	0	Human mouth
	KpM2	0	Human mouth
*Klebsiella alba*	KCTC 12878^T^	4	KCTC
*Klebsiella michiganensis*	LB8	1	Pond water
	LB17	1	Pond water
	NB18	2	Pond water
*Raoultella ornithinolytica*	BB3	2	Pond water
	BB4	1	Pond water
	LB19	3	Pond water
	LB20	3	Pond water
	NB17	3	Pond water
*Raoultella planticola*	LB13	1	Pond water
*Escherichia coli*	MG 1655	1	Lab strain
*Acinetobacter baumannii*	KCTC 2508^T^	0	KCTC
*Pseudomonas aeruginosa*	KCTC 1750^T^	0	KCTC
*Staphylococcus aureus*	KCTC 1621	0	KCTC

This table shows a list of bacterial strains tested for host range test. Lysis scoring: 4, complete clearing; 3, clearing throughout but with faintly hazy background; 2, substantial turbidity throughout the cleared zone; 1, a few individual plaques; 0, no clearing.

### 2.2 Phage isolation and purification

Phage K14-2 was isolated from a water sample collected from the Jungnangcheon River (37.553267, 127.043876) located in Seoul, Republic of Korea. For phage isolation, the sample was centrifuged at 8,000 × *g* for 5 min at 4°C and then filtered through 0.45 and 0.22 μm PVDF syringe filters (GVS) to remove sediments and bacterial cells. Subsequently, the double-layer agar (DLA) method was used to form and isolate plaques. Briefly, 10 μl of the filtrate and 500 μl of an exponential phase of *K. pneumoniae* KCTC 12385*^T^* culture were mixed in a 1.5 ml tube. The mixture was incubated at 37°C for 10 min to allow phage adsorption to the bacterial cells. After incubation, the mixture was added to 4.5 ml of 0.5% soft agar LB medium and overlaid onto a 1.5% hard LB agar plate. Both the soft and hard agars were supplemented with 5 mM CaCl_2_. After overnight incubation at 37°C, the resulting plaques were isolated using sterile pipette tips and stored in SM buffer (100 mM NaCl, 8 mM MgSO_4_⋅7H_2_O, 50 mM Tris-Cl, pH 7.5). Three consecutive isolation procedures were performed to ensure the pure isolation of a single phage plaque. The isolated phage was stored at 4°C for future studies.

### 2.3 Multiplicity of infection, killing curve assay, and one-step growth curve

To establish optimal conditions for further experiments, the MOI between *K. pneumoniae* KCTC 12385*^T^* and phage K14-2 was determined. MOI refers to the ratio of plaque-forming units (PFUs) to colony-forming units (CFUs). Phage and bacterial titers were obtained using the DLA method and colony counting method, respectively. Liquid host cultures were mixed with the appropriate concentration of the phage to achieve MOIs of 100, 10, 1, 0.1, 0.01, and 0.001 (Table 2). The mixed cultures were incubated at 37°C for 10 min to allow phage adsorption. The optimal MOI was determined based on the highest phage titer released from the mixture.

**TABLE 2:** Optimal MOI of the phage K14-2.

Tested MOI	Initial concentration		Final phage titer (PFU/ml)
	Phage (PFU/ml)	Host (CFU/ml)	
100	4 × 10^8^	4 × 10^6^	1.85 × 10^9^
10	4 × 10^8^	4 × 10^7^	1.21 × 10^9^
1	4 × 10^8^	4 × 10^8^	6.29 × 10^10^
**0.1**	**4 × 10^7^**	**4 × 10^8^**	**1.06 × 10^11^**
0.01	4 × 10^6^	4 × 10^8^	7.71 × 10^10^
0.001	4 × 10^5^	4 × 10^8^	6.88 × 10^10^

This table shows the number of plaques formed with the MOI. Values written in bold indicates the optimal values of the MOI.

Additionally, a killing curve assay was conducted to assess the bacterial inhibition effect of phage K14-2. The method used was described by Das and Kaledhonkar (2024). A 100 μl aliquot of freshly incubated host bacterial culture and 100 μl of phage were mixed to achieve an MOI of 0.1 in 96-well plates. LB broth and uninfected host cultures were included as blank and control, respectively, then the plates were incubated at 37°C. The optical density of the bacterial cell was measured at 600 nm every 30 min for 300 min using a Synergy MX spectrophotometer (BioTek).

A one-step growth curve was plotted to determine the incubation period and burst size of phage K14-2, following the method described by Kropinski (2018) with minor adjustments. Briefly, mid-log phase bacteria (OD_600_ = 0.4) in liquid culture were mixed with 1 × 10^7^ PFU/ml of phage and incubated at 37°C for 10 min for phage adhesion. The mixture was serially transferred to three flasks containing LB broth, resulting in 10^–2^, 10^–3^, and 10^–4^ dilutions. The flasks were incubated in a 37°C water bath. Every 10 min, 0.1 ml of the cultures was removed from each flask, added to soft agar, and then overlaid onto hard agar plates. The plates were incubated overnight at 37°C, and the number of plaques was counted to plot the growth curve.

### 2.4 Physical stability

The sensitivity of phage K14-2 to thermal and pH stresses was evaluated using the method described by Das and Kaledhonkar (2024). For thermal stability, phage suspensions at a concentration of 1 × 10^10^ PFU/ml were incubated in a water bath at 40, 50, 60, 70, and 80°C for 60 min. Phage titers were then determined using the DLA method. Similarly, pH stability was assessed by incubating phage suspensions of the same titer in SM buffer adjusted to pH levels ranging from 1 to 14 for 60 min, followed by their measurement.

### 2.5 Host range assay

The host range of phage K14-2 was assessed using a spot test with 21 bacterial strains listed in Table 1. For the spot test, bacterial cultures were mixed with soft agar and overlaid onto agar plates to solidify. Subsequently, 10 μl of phage suspensions were spotted on to the surface of the plates. After overnight incubation at 37°C, plaque formation was observed. The plaques were scored according to the system described by Kutter (2009), as follows: 4, complete clearing; 3, clearing with a faintly hazy background; 2, turbidity throughout the cleared zone; 1, a few individual plaques; 0, no plaque formation. The host range assay was repeated three times for each bacterial strain tested.

### 2.6 Transmission electron microscopy

The morphological characteristics of phage K14-2 were observed using a transmission electron microscope (TEM). A 10 μl drop of 1 × 10^10^ PFU/ml purified phage suspension was placed on a Formvar film (EMS) and stained with 2% (w/v) phosphotungstic acid (PTA) for 5 min. The stained film was observed with JEM1010 TEM (JEOL) at an accelerating voltage of 80 kV.

### 2.7 Phage K14-2 DNA extraction and genome sequencing

Genomic DNA of phage K14-2 was extracted from a 1 × 10^10^ PFU/ml purified phage suspension using the DNeasy Blood and Tissue Kit (Qiagen) with additional procedures. The phage suspension was first mixed with RDD buffer, DNase I, and RNase A, and incubated at 37°C for 90 min to eliminate external DNA or RNA. Following this, 0.5M EDTA and Proteinase K were added, and the mixture was incubated for an additional 90 min at 56°C. The remaining steps followed the manufacturer’s protocol. For library preparation, the Illumina TruSeq Nano DNA (350) library was utilized. Genome sequencing was performed using the Illumina NovaSeqX platform (Illumina). Prior to assembly, the quality of the raw sequences was assessed using FastQC v0.11.7 software,^1^ and adaptor sequences were removed with Trimmomatic v0.38 (Bolger et al., 2014). The whole phage genome was assembled using SPAdes v3.15.0 (Bankevich et al., 2012).

### 2.8 Genome annotation

The annotation of the K14-2 phage whole genome was performed using Prokka v1.14.6 (Seemann, 2014) and the Rapid Annotation using Subsystem Technology (RAST) server^2^ (Brettin et al., 2015). To identify closely related *Klebsiella* phages, a BLASTn^3^ (Altschul et al., 1997) search against the NCBI database was utilized. The whole genome map was constructed using the CGView Server.^4^ Potential tRNA genes within the phage genome were identified using tRNAscan-SE (Chan and Lowe, 2019). Two similar phage genome sequences were analyzed for linear comparison with Easyfig program (Sullivan et al., 2011).

### 2.9 Phylogenetic analysis

To construct a phylogenetic tree, amino acid sequences of the major capsid protein (MCP) from closely related phages were retrieved using the BLASTp program with the NCBI database. These sequences were then aligned using the CLUSTAL W program in BioEdit v5.0.9 software. The aligned sequences were subjected to phylogenetic tree reconstruction using the MEGA v7.0.26 program, employing the NJ algorithm with 1,000 bootstrap replications.

### 2.10 Data presentation

All data presented in the graphs were generated using GraphPad Prism v8.4.3 software. Error bars represent the standard error of the mean.

## 3 Results

### 3.1 Isolation and morphology of phage K14-2

**FIGURE 1:**
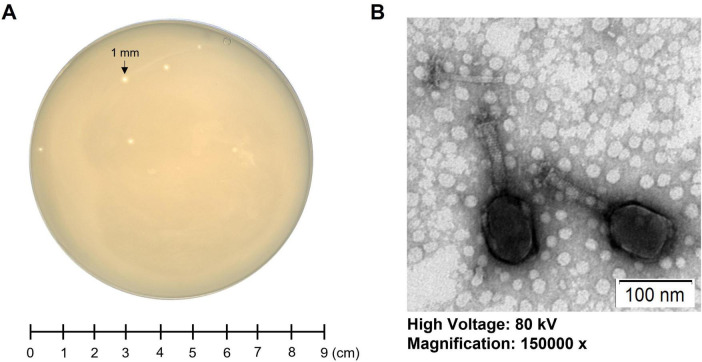
Morphology of phage K14-2. **(A)** Single-plaque morphology of phage K14-2 formed using the double-layer agar (DLA) method after 24 h of incubation. **(B)** Virion morphology of phage K14-2 observed through transmission electron microscopy (TEM).

A novel lytic *Klebsiella* phage, K14-2, was isolated from a river water sample using *K. pneumoniae* as the host bacterium. After incubating the sample for 24 h at 37°C, the plaque morphology was observed, revealing clear, round plaques with diameters of 1–2 mm (Figure 1A). The morphology of the phage virion was visualized using TEM. The phage exhibited an icosahedral head (90.9 ± 3.8 nm), a long sheath (84.0 ± 0.9 nm), and a contractile tail (Figure 1B). The morphological characteristics indicate that the phage resembles features typical of myovirus.

### 3.2 Optimal MOI, killing curve, and one-step growth curve

**FIGURE 2:**
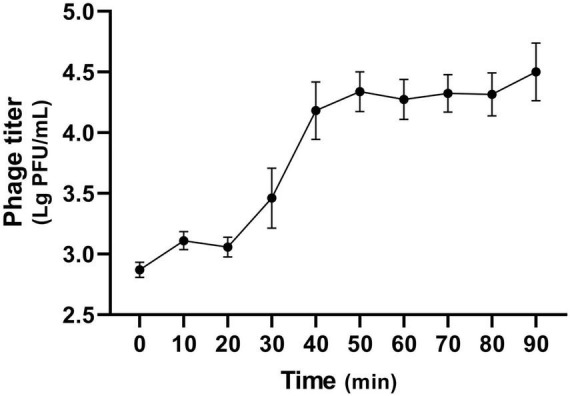
One-step growth curve of K14-2. The one-step growth curve of phage K14-2 incubated at 37°C. Phage titers per infected cells are plotted every 10 min with titers expressed as log PFU values.

The optimal MOI for phage K14-2 was determined to identify the most effective ratio of host to phage for infection. An MOI of 0.1, which resulted in the highest phage titer, was established as the optimal ratio (Table 2). The lytic activity of phage K14-2 was assessed using a killing curve assay (Supplementary Figure 1). The result showed that the phage inhibited the growth of the host bacteria and maintained the host concentration for 5 h, indicating that the growth of the *K. pneumoniae* was effectively inhibited, maintaining levels close to the initial state. Subsequently, a one-step growth curve was generated to determine the burst size and latent period of phage infection. The curve indicated a latent period was approximately 20 min and an outbreak period of approximately 50 min. The burst size of the phage was estimated to be an average of 32.9 PFU/cell, with a standard deviation of 13.3 (Figure 2).

### 3.3 Stability assay

**FIGURE 3:**
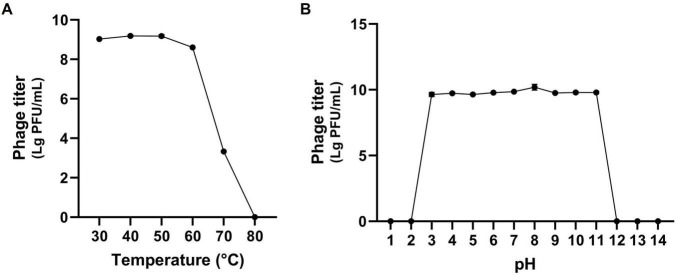
Stability test of K14-2. Tolerance graph of phage K14-2 to temperature and pH stresses. Phage titers per infected cell are plotted as log PFU values. **(A)** Temperature stability assessed across a range of 30–80°C. **(B)** pH stability assessed across a range of pH 1–14.

Phages used in phage therapy should possess not only a broad host range but also the ability to maintain activity under diverse environmental conditions. Specifically, phage therapy intended for humans must be capable of withstanding fluctuations in body temperature and pH levels. Therefore, stability of phage K14-2 with respect to environmental factors, including temperature and pH, was evaluated. The temperature stability assay examined the phage’s resilience at temperatures of 30, 40, 50, 60, 70, and 80°C for 60 min. The assay revealed that the phage was stable at temperatures ranging from 30 to 70°C; however, its lytic activity significantly decreased at 70°C and was completely lost at 80°C (Figure 3A). The pH stability assay assessed the phage’s resilience across a pH range of 1 to 14 for 60 min. The results indicated that the phage retained lytic activity between pH 3 and 11, whereas no activity was observed at pH 1, 2, 12, 13, and 14 (Figure 3B). Collectively, similar to other *Klebsiella* phages, K14-2 was able to maintain activity between 30 and 70°C and pH 3–11 for 60 min. These findings suggest that K14-2 can be used in various environmental conditions, including those found in the human body. However, the assay was limited to a 60 min short incubation time, and further experiments are needed to assess its ability over longer durations.

### 3.4 Host range of K14-2

Before the host range test, the relationships between the host bacterial species were identified using phylogenetic trees reconstructed with 16S rRNA gene sequences and bacterial core gene set extracted from the NCBI reference genomes (Supplementary Figures 2, 3). The resulting trees showed that the species of genus *Raoultella* clustered within the genus *Klebsiella*, indicating a close relationship between two genera. Thus, the host range of phage K14-2 was tested against 10 species and a total of 21 different bacterial strains, as detailed in Table 1. The results demonstrated that phage K14-2 show clearings to various species within the *Klebsiella* and *Raoultella* genera, including *K. pneumoniae* subsp. *pneumoniae*, *K. pneumoniae* subsp. *ozaenae*, *K. alba*, *K. michiganensis*, *R. ornithinolytica*, and *R. planticola*. Additionally, *E. coli*, another member of the Enterobacteriaceae family, exhibited cleared zone with turbidity. In contrast, other ESKAPE pathogens such as *A. baumannii*, *P. aeruginosa*, and *S. aureus* showed no lysis activity.

### 3.5 Genome features of K14-2

**FIGURE 4:**
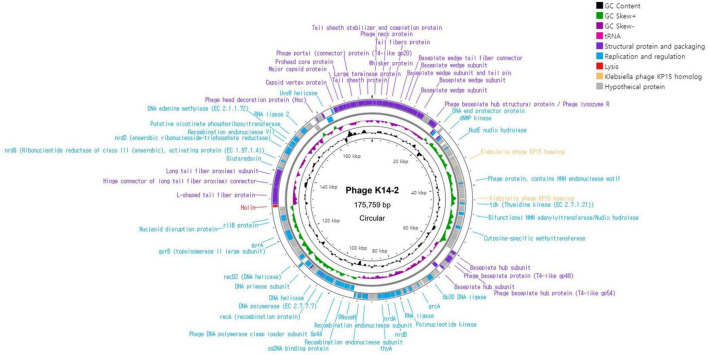
Genome map of K14-2. Visualization of the phage genome, consisting of 175,759 bp and 280 predicted functional genes, using CGView Server. The GC content and GC skew are also presented. Annotated phage genes are categorized into three groups: structural proteins and packaging (purple), replication and regulation (blue), and lysis genes (red).

The complete genome of phage K14-2 was circular double-stranded DNA (dsDNA) molecule comprising 175,759 bp with a GC content of 41.8% (Figure 4). The annotated genome revealed 280 predicted protein-coding genes, including 184 hypothetical proteins. Of these, 96 functional protein-coding genes were classified into three distinct functional modules. Specifically, 41 genes were associated with structural proteins and the packaging system, 54 genes with replication and regulation, and 1 gene with lysis. The structural proteins predominantly included genes encoding the phage capsid, baseplate, sheath, and tail encoding genes. Genes related to replication and regulation encode proteins such as HNH endonuclease motif harboring protein, ligases, DNA polymerases, and helicases. The search for RNA genes within the phage genome revealed two tRNA genes. One of these tRNA genes encodes methionine and was conserved in 72 other phages with 100% similarity. The other tRNA gene is a pseudo type tRNA gene encoding leucine with an anticodon of CAA. Similar sequences of this predicted pseudogene were found in 73 phages, predominantly *Klebsiella* phages. The gene responsible for host lysis was annotated as holin, a gene commonly found in dsDNA phages. Holin encodes pore-forming proteins that create holes in the bacterial cell wall, releasing the assembled virions at the end of the lytic cycle. In contrast, genes associated with the lysogenic cycle, such as integrase, were not identified, indicating that phage K14-2 is a lytic phage. The activity of the lytic gene was further confirmed by the formation of the transparent plaques.

### 3.6 Comparative genomic analysis

**FIGURE 5:**
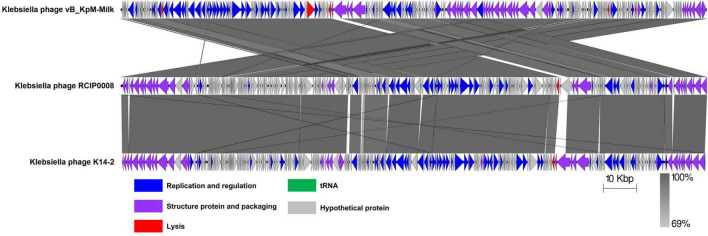
Linear genome comparison of K14-2 and closely related phages. Genome sequences of phages K14-2, vB_KpM-Milk, and RCIP0008 were analyzed for linear comparison using Easyfig software. The position and the transcription direction of the annotated genes were shown by arrows. The arrows were colored according to the gene functions.

Homologous sequences of the phage K14-2 genome were identified using the BLASTn program. The results showed that the phage genome is highly similar to other *K. pneumoniae* phages within the *Slopekvirus* genus. The genome sequence with the highest similarity was that of *Klebsiella* phage vB_KpM-Milk, showing 98.66% similarity and 96% query coverage. According to the species demarcation criteria proposed by the Bacterial Viruses Subcommittee of International Committee on Taxonomy of Viruses (ICTV), novel bacteriophage species should have a similarity lower than 95% (Turner et al., 2021). Using the BLASTn program, the similarity value can be calculated by multiplying the percentage identity by the percentage coverage. Thus, the similarity value between phage K14-2 and vB_KpM-Milk is 94.71%, indicating that phage K14-2 represents a novel species within the *Slopekvirus* genus. Linear genome comparison was also performed with K14-2, vB_KpM-Milk and RCIP0008 phage genomes, showing that K14-2 was highly homologous with other two phages (Figure 5).

### 3.7 Phylogenetic analysis

**FIGURE 6:**
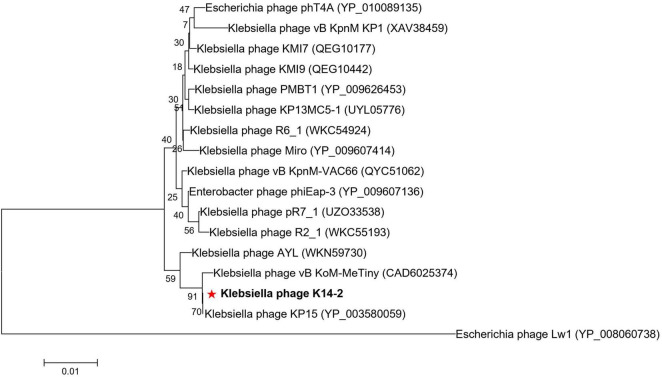
Phylogenetic tree of K14-2 and closely related phages. Phylogenetic tree constructed using the major capsid (MCP) protein sequences of phage K14-2 and other closely related phages. The MCP protein sequences similar to K14-2 were aligned, and the tree was constructed using the neighbor-joining method with 1,000 bootstrap replicates. The symbol “*” indicate the positioning of the phage studied in this paper.

A phylogenetic tree was constructed using the MCP amino acid sequences to identify the evolutionary relationship of phage K14-2 with closely related species (Figure 6). The tree confirmed that phage K14-2 clusters with *Klebsiella* phages within the genus *Slopekvirus* of the family Straboviridae.

## 4 Discussion

As the threat of AMR bacteria to global health continues to rise, efforts to combat bacterial infections through phage therapy have increased significantly (Kviatcovsky et al., 2023; Strathdee et al., 2023). However, the limitations of using a single phage strain with a specific host strain for treatment are well-documented (MacNair et al., 2024). Most of the characterized *Klebsiella* phages exhibit a strain-specific, narrow host range, which limits their application and may contribute to the development of phage resistance in target bacteria. To advance phage therapy research, a comprehensive database of lytic phages with diverse host ranges is necessary to identify optimal therapeutic strategies. Thus, the characterization of diverse phage strains is essential not only for the advancement of phage therapy studies but also for enhancing our understanding of phage taxonomy.

In this study, a novel lytic phage, K14-2, belonging to the genus *Slopekvirus*, was isolated from a river sample targeting *K. pneumoniae*. The biological and genomic characteristics of the phage were examined. The biological characteristics of phage K14-2, including its morphology, MOI, killing curve, one-step growth curve, and stability, were similar to those of other closely related *Klebsiella* phages. However, the phage exhibited a relatively broad host range, as it was capable of infecting 14 strains within the *Klebsiella* and *Raoultella* genera, as well as a strain of *E. coli* within the Enterobacteriaceae family. Cleared zones were observed in *K. pneumoniae* subspecies, *K. alba*, *K. michiganensis*, *R. ornithinolytica*, and *R. planticola*. Although the *Raoultella* genus is distinguished from *Klebsiella* by distinct *gyrB* sequences, 16S rRNA gene sequence and whole-genome-based phylogenetic analyses from this study suggest that species within the genus *Raoultella* still closely align with *Klebsiella* (Wyres et al., 2020). This genomic similarity between the two genera may explain the overlap in the phage host range. Additionally, K14-2 exhibited clearings with turbidity against *E. coli*, suggesting that the phage may utilize membrane proteins commonly found in the Enterobacteriaceae family.

Further analysis of the genomic characteristics of phage K14-2 indicated that the phage possesses three types of functional proteins. Gene annotation revealed that approximately 15% of the genes are related to structural proteins and packaging, 19% to replication and regulation, and 66% to functions that remain unknown. The lysis-related gene holin, commonly found in the Caudoviricetes class, was identified, while lysogeny-related genes were absent, indicating that K14-2 is a virulent phage. A methionine tRNA and a hypothetical tRNA were identified in the genome. The presence of these tRNAs is suggested to be related to the phage’s lytic activity (Bailly-Bechet et al., 2007); however, their exact roles remain poorly understood and require further investigation. The genome of K14-2 showed the highest similarity to the phage vB_KpM-Milk, with other similar phages predominantly belonging to the genus *Slopekvirus* within the Straboviridae family. Phylogenetic analysis based on MCP amino acid sequences revealed the evolutionary lineage of K14-2 alongside other *Klebsiella* phages.

Collectively, the biological and genomic characteristics of K14-2 suggest that it is a novel *Slopekvirus* phage with a wide host range against *Klebsiella* and *Raoultella* genera. To address the limitations of phage therapy associated with a narrow host range, various approaches, including the use of phage cocktails and the engineering of phage tails, are currently under exploration (Federici et al., 2022; Gencay et al., 2024; Yehl et al., 2019). The broad lytic activity of K14-2 demonstrated in this study implies its potential applicability across a wide range of species. Future studies are needed to identify the specific infection receptors involved.

## 5 Conclusion

In this study, we isolated a lytic phage, K14-2, from a river water sample using *K. pneumoniae* as the host. Screening of its biological and genomic characteristics revealed a high degree of similarity to other *Klebsiella* phages within the genus *Slopekvirus* of the Straboviridae family. The phage demonstrated a relatively broad host range, capable of infecting *Klebsiella* and *Raoultella* genera. Its notable environmental stability suggests that this phage could be a valuable candidate for future studies on phage therapy.

## Data Availability

The datasets presented in this study can be found in online repositories. The names of the repository/repositories and accession number(s) can be found in this article/Supplementary material. The GenBank accession number for the phage K14-2 whole genome is PP978606.

## Footnotes

1
http://www.bioinformatics.babraham.ac.uk/projects/fastqc

2
https://rast.nmpdr.org/

3
https://blast.ncbi.nlm.nih.gov/Blast.cgi

4
https://cgview.ca/
