# Supplementary material for: Isolation and characterization of a novel lytic phage K14-2 infecting diverse species of the genus Klebsiella and Raoultella
Source: Front Microbiol. 2025 Jan 17;15:1491516. doi: 10.3389/fmicb.2024.1491516 (PMC11782208; doi:10.3389/fmicb.2024.1491516)

Supplementary data

Isolation and characterization of a novel lytic phage K14-2 infecting diverse species of the genus *Klebsiella* and *Raoultella*.

Seomin Kang[1], Jeong-Eun Han[1], Young-Sik Choi[1], In-Chul Jeong[1], and Jin-Woo Bae[1,2,*]

[1] Department of Biology, College of Science, Kyung Hee University, Seoul 02447, Republic of Korea.

[2] Department of Biomedical and Pharmaceutical Sciences, and Department of Life and Nanopharmaceutical Sciences, Kyung Hee University, Seoul 02447, Republic of Korea

* For correspondence: Jin-Woo Bae.

Tel.: +82-2-961-2312. Fax: +82-2-961-9155. E-mail: baejw@khu.ac.kr

Running title: Characterization of a *Klebsiella* phage

Key words: Bacteriophage, phage, *Klebsiella pneumoniae*, *Klebsiella*, *Raoultella*, *Slopekvirus*

Supplementary figure legends

Supplementary data Figure S1. A killing curve of the phage K14-2

A killing curve demonstrating the killing efficiency of phage K14-2 against the host *K. pneumoniae* KCTC 12385^{T}. LB broth was used as the blank, and pure bacterial culture was used as the control.

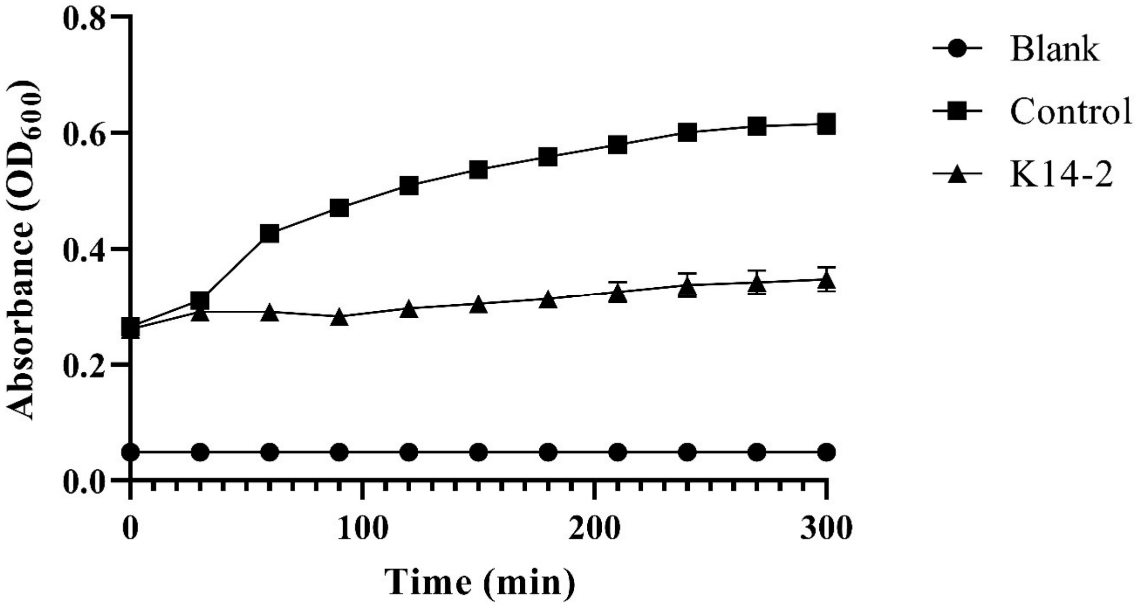

Supplementary data Figure S2. A phylogenetic tree based on 16S rRNA gene sequences of the strains used for host range test

A phylogenetic consensus tree based on the 16S rRNA gene sequences of the bacterial species used for the host range test. The tree displays the relationship between the tested species. The tree construction was performed using the NJ algorithm, then the reliability of the clades was validated with ML and MP algorithms. The numbers at the nodes show bootstrap values (NJ.ML/MP) as percentages of 1000 replicates and the values lower than 70% are not shown. The filled diamonds show the identical branches from three algorithms. *Escherichia coli* NBRC 102203^{T} was used as an outgroup. Bar, 0.005 accumulated changes per nucleotide.

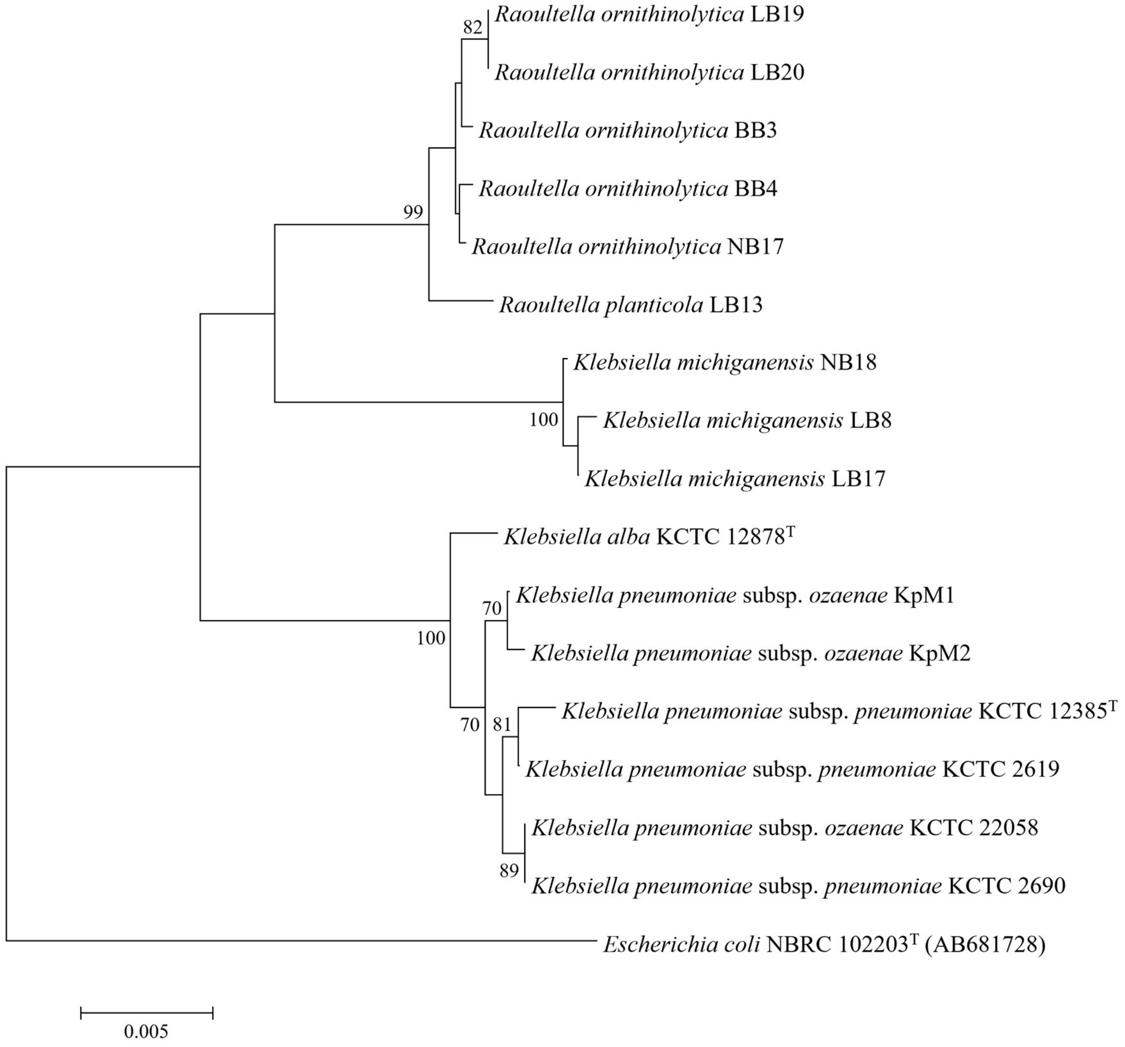

Supplementary data Figure S3. A UBCG tree based on a bacterial core gene set of species in genera *Klebsiella* and *Raoultella*

A phylogenetic consensus tree based on a core bacterial gene set of species in genera *Klebsiella* and *Raoultella*. The tree displays the relationship between the genera *Klebsiella* and *Raoultella*. The type species of the genera *Citrobacter*, *Cronobacter*, *Enterobacter*, *Escherichia*, *Kluyvera*, and *Leclercia*, from *Enterobacteriaceae* were also included. The numbers at the nodes show the Gene Support Index, the numbers of single-gene trees that support the branch. Bar, 0.02 accumulated changes per nucleotide.

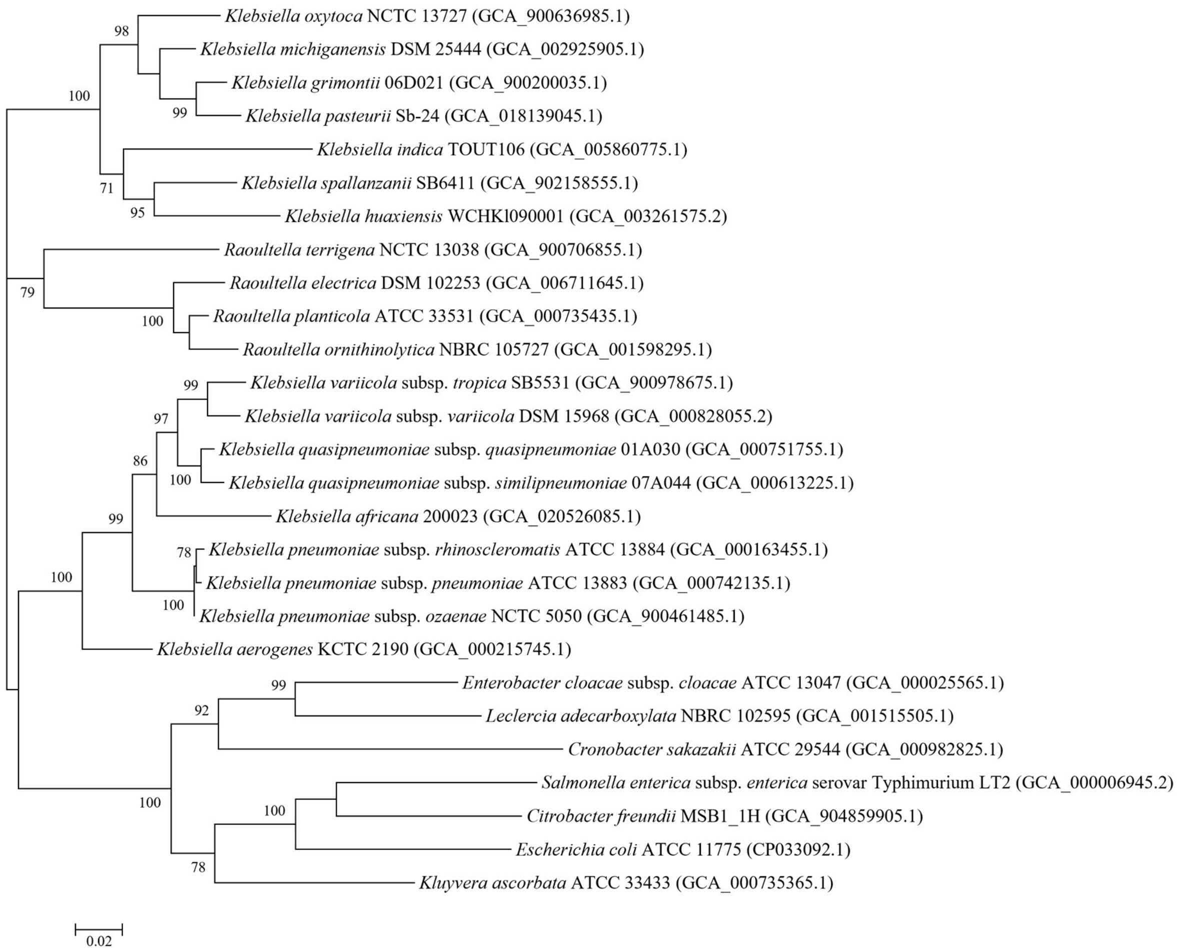

Supplement: Supplementary file 1 [file Data_Sheet_1.pdf]
